# Germline Variants of *CYBA* and *TRPM4* Predispose to Familial Colorectal Cancer

**DOI:** 10.3390/cancers14030670

**Published:** 2022-01-28

**Authors:** Lizhen Zhu, Beiping Miao, Dagmara Dymerska, Magdalena Kuswik, Elena Bueno-Martínez, Lara Sanoguera-Miralles, Eladio A. Velasco, Nagarajan Paramasivam, Matthias Schlesner, Abhishek Kumar, Ying Yuan, Jan Lubinski, Obul Reddy Bandapalli, Kari Hemminki, Asta Försti

**Affiliations:** 1 Division of Molecular Genetic Epidemiology, German Cancer Research Center (DKFZ), Im Neuenheimer Feld 580, D-69120 Heidelberg, Germany; azhulijane@163.com (L.Z.); b.miao@kitz-heidelberg.de (B.M.); abhishek.abhishekkumar@gmail.com (A.K.); a.foersti@kitz-heidelberg.de (A.F.); 2 Department of Medical Oncology, The Second Affiliated Hospital of Zhejiang University School of Medicine, Hangzhou 310009, China; yuanying1999@zju.edu.cn; 3 Hopp Children’s Cancer Center (KiTZ), D-69120 Heidelberg, Germany; 4 Division of Pediatric Neurooncology, German Cancer Research Center (DKFZ), German Cancer Consortium (DKTK), D-69120 Heidelberg, Germany; 5 Department of Genetics and Pathology, Hereditary Cancer Center, Pomeranian Medical University, Unii Lubelskiej 1, 71-252 Szczecin, Poland; dagmara.dymerska@gmail.com (D.D.); magdalenakuswik@gmail.com (M.K.); lubinski@pum.edu.pl (J.L.); 6 Splicing and Genetic Susceptibility to Cancer, Instituto de Biología y Genética Molecular (CSIC-UVa), 47003 Valladolid, Spain; buenome@usal.es (E.B.-M.); lara.sanoguera@alumnos.uva.es (L.S.-M.); eavelsam@ibgm.uva.es (E.A.V.); 7 Computational Oncology, Molecular Diagnostics Program, National Center for Tumor Diseases (NCT), D-69120 Heidelberg, Germany; n.paramasivam@dkfz-heidelberg.de; 8 Bioinformatics and Omics Data Analytics, German Cancer Research Center (DKFZ), D-69120 Heidelberg, Germany; matthias.schlesner@informatik.uni-augsburg.de; 9 Institute of Bioinformatics, International Technology Park, Bengaluru 560066, India; 10 Manipal Academy of Higher Education (MAHE), Manipal 576104, India; 11 Medical Faculty Heidelberg, Heidelberg University, D-69120 Heidelberg, Germany; 12 Faculty of Medicine and Biomedical Center in Pilsen, Charles University in Prague, 30605 Pilsen, Czech Republic; 13 Division of Cancer Epidemiology, German Cancer Research Center (DKFZ), D-69120 Heidelberg, Germany

**Keywords:** whole-genome sequencing, cancer predisposition, mucin, reactive oxygen species

## Abstract

**Simple Summary:**

Whole-genome sequencing and bioinformatics analysis on unique colorectal cancer families revealed two attractive candidate predisposition genes, *CYBA* and *TRPM4*, each with a loss-of-function variant. Supported by our functional studies, we suggest that the two gene defects mechanistically involve intestinal barrier integrity through reactive oxygen species and mucus biology, which converges in chronic bowel inflammation, a known risk factor for colorectal cancer.

**Abstract:**

Familial colorectal cancer (CRC) is only partially explained by known germline predisposing genes. We performed whole-genome sequencing in 15 Polish families of many affected individuals, without mutations in known CRC predisposing genes. We focused on loss-of-function variants and functionally characterized them. We identified a frameshift variant in the *CYBA* gene (c.246delC) in one family and a splice site variant in the *TRPM4* gene (c.25–1 G > T) in another family. While both variants were absent or extremely rare in gene variant databases, we identified four additional Polish familial CRC cases and two healthy elderly individuals with the *CYBA* variant (odds ratio 2.46, 95% confidence interval 0.48–12.69). Both variants led to a premature stop codon and to a truncated protein. Functional characterization of the variants showed that knockdown of *CYBA* or *TRPM4* depressed generation of reactive oxygen species (ROS) in LS174T and HT-29 cell lines. Knockdown of *TRPM4* resulted in decreased MUC2 protein production. *CYBA* encodes a component in the NADPH oxidase system which generates ROS and controls, e.g., bacterial colonization in the gut. Germline *CYBA* variants are associated with early onset inflammatory bowel disease, supported with experimental evidence on loss of intestinal mucus barrier function due to ROS deficiency. *TRPM4* encodes a calcium-activated ion channel, which, in a human colonic cancer cell line, controls calcium-mediated secretion of MUC2, a major component of intestinal mucus barrier. We suggest that the gene defects in *CYBA* and *TRPM4* mechanistically involve intestinal barrier integrity through ROS and mucus biology, which converges in chronic bowel inflammation.

## 1. Introduction

Colorectal cancer (CRC) is the third most commonly diagnosed cancer and the second leading cause of death from cancer [1]. Familial CRC accounts for some 15% of all cases, and the twin estimate of heritability amounts up to 30%, but only 2–5% of CRCs are confirmed to be caused by inherited syndromes related to CRC [2,3,4,5,6]. Among these, Lynch-syndrome-related CRC, caused by germline mutations in mismatch repair (MMR) genes *MLH1*, *MSH2*, *MSH6*, *PMS2* and *EPCAM*, accounts for the majority of hereditary CRC. These tumors are characterized by deficient MMR (dMMR). Rarer syndromes are related to mutations in genes *APC*, *MUTYH*, *STK11*, *PTEN*, *BMPR1A* and *SMAD4* [6].

With the advancement of next-generation sequencing technologies, increasing number of genes have been reported to be possible candidates for CRC predisposition. These include *POLE*, *POLD1*, *NTHL1*, *GERM1*, *GALNT12*, *RNF43*, *RPS20*, *MLH3* and *MSH3* [3,6,7,8,9]. According to the National Comprehensive Cancer Network clinical practice guidelines, most of these new candidates do not have well-established evidence of increased risk for CRC [3]; however, with more data, some of these genes will be proved to be CRC predisposing genes [3,8,10]. Epidemiological evidence, such as lacking correlation of CRC risk between spouses, suggests that most of familial aggregation in this cancer is genetic [11]. Thus, it is likely that novel predisposing genes will be identified in families of affected individuals.

Gut homeostasis is maintained by a physical separation of the microbial community from the gut epithelium by a mucus barrier. MUC2 mucin, expressed in the goblet cells of the intestine, is the main component of the intestinal mucus barrier; its expression is commonly lost in CRC, and its loss predicts adverse outcome [12]. MUC5AC and MUC6 are rarely observed in the normal colon, but their expression in CRC is associated with favorable outcome. MUC1 is expressed on the apical surface of most epithelial cells, and it does not seem to affect the outcome.

Most ‘classical’ cancer-predisposing genes were found in linkage studies in families with multiple affected patients [13]. Following this paradigm, we performed germline whole-genome sequencing (WGS) in 15 Mendelian type of CRC families unrelated to known CRC-predisposing genes, focusing on loss-of function variants. We identified a frameshift variant in *CYBA* in one family and a splice site variant in *TRPM4* in another family; while the genes encode proteins in diverse pathways, their functions appeared to converge in intestinal-barrier integrity and mucus-biology-targeting inflammatory bowel disease, a known risk factor of CRC [14].

## 2. Materials and Methods

### 2.1. Population Recruitment

In several regions of Poland, population screening was performed mainly in the years 2000–2014, in which questionnaires about cancer family history were collected. Persons with positive CRC family history were invited to genetic outpatient clinics all over Poland, and their more detailed family histories were collected through face-to-face detailed interviews. Similarly, persons with negative cancer family history were interviewed. An average review took 20–30 min. Eligible individuals were asked to participate in the study, and they signed an informed consent form.

### 2.2. Colorectal Cancer Families

Fifteen families with strong CRC aggregation compatible with an autosomal dominant pattern of inheritance were recruited to the study. Each family had at least three pathologically confirmed CRC cases; 13 families had at least one case diagnosed below the age of 55 years. In Poland, colonoscopy is offered for the family members, so many families also had members diagnosed with polyps. Twelve families had CRC in at least two generations. In the other three families, one had 5 siblings diagnosed with CRC and 5 of their children had polyps at the age of 41–57 years. In another family, 3 siblings were diagnosed with CRC at the age of 53, 54 and 63 years; their father had been diagnosed with prostate cancer and had died at the age of 72 years and their children were born in 1980s and thus uninformative regarding the CRC status. The third family had 3 siblings diagnosed with CRC at the age of 50, 55 and 62 years; their father had suffered from cancer and died at the age of 55 years. All families were screened for alterations in *APC*; the mismatch repair genes *MLH1, MSH2* and *MSH3*; and large deletions in *EPCAM* and *POLE* p.Leu424Val, *POLD1* p.Ser478Asn and *NTHL1* p.Gln90* mutations and were found to be negative.

### 2.3. Validation Cohort

Altogether, 1705 unrelated familial CRC cases and 1674 healthy elderly individuals without a family history of cancer were included in the validation cohort.

### 2.4. DNA Isolation

Peripheral blood samples were collected from affected and unaffected family members who agreed to participate in the study, as well as from the validation cohort. Genomic DNA was isolated by using a modified Lahiri and Schnabel method [15].

### 2.5. Germline Whole-Genome Sequencing (WGS)

WGS was performed in the Illumina X10 platform, using DNA extracted from the blood samples as paired-end sequencing with a read length of 150 bp. Mapping of reads to the human reference genome (GRCh37 assembly version hs37d5) was performed by using BWA mem (version 0.7.8), and duplicates were marked by using Picard (version 1.125). Small variants, single nucleotide variants (SNVs) and indels were called by using Platypus (version 0.8.1). Variants were annotated by using ANNOVAR [16], dbNSFP v2.9 [17], 1000 Genomes phase III [18], dbSNP [19] and ExAC [20], as described previously [21]. On the variants that passed all the internal Platypus filters, we performed a further filtering while considering the QUAL score >20 and coverage of minimum 5 reads. The sequencing coverage and quality statistics for each sample are summarized in Supplementary Table S1. Minor allele frequency (MAF) of 0.1% was used with respect to population databases (the 1000 Genomes phase III [18] and non-TCGA ExAC [20] data), and the variant frequency of 5% from the local datasets was used to remove technical artefacts. Pairwise comparison of variants among the cohort was performed to check for sample swaps and family relatedness.

### 2.6. Variant Identification

Variants were filtered based on the pedigree information, considering the members diagnosed with CRC as cases. Family members who were diagnosed with polyps were considered possible variant carriers, as were individuals whose parents were diagnosed with CRC and who had not yet reached the age of diagnosis of the youngest CRC case in the family. Unaffected family members were considered as controls. All variants segregating with the disease were filtered for loss-of-function (stop gain, frameshift and splicing) variants. All variants with a MAF of <0.1% in the gnomAD (https://gnomad.broadinstitute.org/ (accessed on 17 January 2022)) non-Finnish European population were further screened for their location in the gene. Variants located in the last exon or in the non-protein coding transcript of the gene were excluded. Sequencing data were visually inspected by using the Integrative Genomic Viewer (IGV [22]) to exclude false positive variants. Combined Annotation-Dependent Depletion (CADD [23]) score was used to evaluate the deleteriousness of the variants; the scores >20 and >30 are indicative of the top 1% and top 0.1% of deleterious variants, respectively. The final variant selection was based on a search of the literature.

### 2.7. Copy Number Variants

Structural variants were analyzed by using the SmallPedigree-WGS workflow of Canvas (version 1.40.0.1613 [24]) separately to detect larger copy number variants as described earlier [25]. Variants that affected known cancer-predisposing genes were manually inspected by using IGV.

### 2.8. Variant Confirmation

Candidate variants and their segregation with the disease in the families were confirmed in all available family members by Sanger sequencing with the primers *CYBA* F 5′-GGAGCTTGGTTTCTCACTTGG-3′, R 5′-GGAGCTCCTCGGATTTGGA-3′; and *TRPM4* F 5′-GTGGCTCTGTGTCCCATAGG-3′, R 5′-TCTACACAGACCCAAACGCT-3′. The variants were checked for frequency in the 1705 familial CRC cases and 1674 healthy elderly individuals, using custom-made Taqman assays, and the existence of the heterozygous variants was confirmed by using the Sanger sequencing.

### 2.9. TRPM4 Minigene Assay

#### 2.9.1. Splicing Predictions

In silico analysis of the wild-type (WT) and mutant sequences was made with the algorithm Max Ent Scan [26] that is integrated into the splicing software of Human Splicing Finder version 3.1 (HSF, http://www.umd.be/HSF3/ (accessed on 17 January 2022)) [27]. Variant and transcript descriptions were according to the Human Genome Variation Society (HGVS) guidelines on the basis of the *TRPM4* GenBank sequence NM_017636.4.

#### 2.9.2. Construction of the Minigene mgTRPM4_ex1-2

A single fragment with exons 1 and 2 of the *TRPM4* gene (728 bp) was amplified from a patient (III-5) DNA with the variant with Kapa High Fidelity polymerase (Kapa Biosystems, Wilmington, MA) and the primers mgTRPM4_ex1-fw 5′ AGTCACCTGGACAACCTCAAAGGCACCTTT GAAGCAGAGCCGGCGGAGGG 3′ and mgTRPM4_ex2-rv 5′ ATAAGCTTGATATCGAATTCCTGCAGCCCG AGCACATAGAAGAGACATCGG 3′ (cloning tails are underlined). This insert has the following structure: EX1 [90 bp]–ivs1 [301 bp]–EX2 [68 bp]–ivs2 [269bp]. This fragment was cloned into the splicing vector pSAD v9.0 (Patent P201231427, CSIC) [28] by using Overlap Extension PCR [29] to generate a chimeric fusion of vector exon V1-*TRPM4* exon 1. Several colonies were sequenced at the Macrogen facility (Macrogen, Madrid, Spain) to confirm the presence of both WT and mutant minigenes (c.25–1G > T).

#### 2.9.3. Transfection of Eukaryotic Cells

Transfections were carried out as previously described [30]. Cells were transfected with 1 µg of each construct, using low-toxicity Lipofectamine (Life Technologies, Carlsbad, CA, USA). To inhibit nonsense mediated decay (NMD), a 4 h incubation with cycloheximide 300 µg/mL (Sigma-Aldrich, St. Louis, MO, USA) was carried out.

#### 2.9.4. Reverse-Transcription PCR of Minigene RNA

RNA was purified with the Genematrix Universal RNA Purification Kit (EURx), including on-column DNAse I digestion. Reverse transcription was carried out with 400 ng of RNA and the RevertAid First Strand cDNA Synthesis Kit (Life Technologies), using the vector-specific primer RTPSPL3-RV (5′-TGAGGAGTGAATTGGTCGAA-3′). Samples were incubated at 42 °C for 1 h, followed by 5 min at 70 °C. Then, 40 ng of cDNA (final volume of 50 µL) was amplified with RT-pMAD502-FW (5′-GAGGTTCTTCGAGTCCTTT-3′) and RTpSAD-RV (Patent P201231427) (full-length transcript 394 nt), using Platinum-Taq DNA polymerase (Life Technologies). Samples were denatured at 94 °C for 2 min, followed by 35 cycles x (94 °C, 30 s; 60 °C, 30 s; 72 °C, 30 s), and a final extension step at 72 °C for 5 min. RT-PCR products were run on 1.2% agarose gels and sequenced at the Macrogen facility.

#### 2.9.5. Capillary Electrophoresis of Fluorescent RT-PCR

In order to relatively quantify all transcripts, semi-quantitative fluorescent RT-PCRs were performed in triplicate with primers RT-pMAD502-FW and FAM-RTpSAD-RV, as described earlier [30].

### 2.10. Cell Culture

Human colorectal adenocarcinoma cell line HT-29 (RRID:CVCL_0320) (a kind gift from Peter Krammer’s lab, DKFZ) was cultured in RPMI 1640 media (21875091, Life Technologies, Carlsbad, CA, USA) supplemented with 10% fetal bovine serum (FBS) (10500064, Life Technologies, Carlsbad, CA, USA). Mucin-secreting colorectal adenocarcinoma cell line LS174T (RRID:CVCL_1384) (C0009013, AddexBio, San Diego, CA, USA) was cultured in RPMI 1640 media supplemented with 10% FBS supplemented with 2 mM L-glutamine (51411C, Sigma-Aldrich, Saint Louis, MO, USA). HEK293T (RRID: CVCL_0063) cells were a gift from Andreas Trump (DKFZ, Heidelberg). The cells were maintained in DMEM high glucose supplemented with 10% FBS (Gibco), penicillin (50 U/mL, Life Technologies, Carlsbad, CA, USA) and streptomycin (50 µg/mL, Life Technologies, Carlsbad, CA, USA). The cells were authenticated by using SNP or STR profiling within the last 3 years, and all experiments were performed with mycoplasma-free cells.

### 2.11. Site-Directed Mutagenesis

pcDNA4TO-HA-*TRPM4* was a generous gift from Hugues Abriel [31]. Gateway clone of pENTR221-*CYBA* was obtained from DKFZ′s genomics and proteomics core facility (GPCF). We used LR Clonase Enzyme Mix (11791-019, Invitrogen, Waltham, MA, USA) to make pENTR221-*CYBA* into pDEST26-*CYBA*. Variants identified in the WGS were introduced into respective plasmids by using QuikChange II XL Site-Directed Mutagenesis Kit (200521, Agilent Technologies, Santa Clara, CA, USA). Mutated cDNA clones were confirmed by Sanger sequencing before using them in further experiments. Plasmids were scaled up by transforming into bacteria and harvested by HiPure Plasmid Midiprep Kit (K210005, Invitrogen, Waltham, MA, USA), and the sequences were checked by Sanger sequencing. Since the *TRPM4* variant was a splice site variant not affecting an exon, we created the pcDNATO-HA-*TRPM4* c.25delAG according to the minigene results of the original *TRPM4* variant. Primers for site directed mutagenesis to get pcDNATO-HA-*TRPM4* c.25delAG and pDEST26-*CYBA* c.246delC were *TRPM4* F 5′-CGGAGAAGGAGCAGCTGGATCCCCAAGA-3′, R 5′-TCTTGGGGATCCAGCTGCTCCTTCTCCG-3′; *CYBA* F 5′-TAGTAATTCCTGGTAAAGGCCCGAACAGCTTCAC-3′, R 5′-GTGAAGCTGTTCGGGCCTTTACCAGGAATTACTA-3′. Sanger sequencing primers for mutated plasmids were *TRPM4* F 5′-CACGCTGTTTTGACCTCCAT-3′, R 5′-CGGAGGAAATTGCTGTGCTT-3′; *CYBA* F 5′-CATGTGGGCCAACGAACAG-3′, R 5′-TCAGTAGGTAGATGCCGCTC-3′.

### 2.12. SiRNA Mediated Knockdown of CYBA and TRPM4

HT-29 and LS174T cells were transfected with human CYBA siRNA (sc-36149, Santa Cruz Biotechnology, INC) to knockdown CYBA or human TRPM4 siRNA (sc-45439, Santa Cruz Biotechnology, Heidelberg, Germany) to knockdown TRPM4, or scrambled siRNA (sc-37007, Santa Cruz Biotechnology, Heidelberg, Germany) as a control. All the siRNA products consist of pools of three to five target-specific 19–25 nt siRNAs designed to knockdown gene expression. HEK293T cells were transfected with wild-type (WT) or mutated (MUT) pDEST26-CYBA or pDEST26 and WT or MUT pcDNA4TO-HA-TRPM4 or pcDNA4TO. All the transfections were performed with Lipofectamine 2000 (11668027, Invitrogen, Waltham, MA, USA).

### 2.13. In Vitro Cell Proliferation Assay

Cell proliferation was estimated by using a cell counting kit 8 (96992-500TESTS-F, Sigma-Aldrich, Saint Louis, MO, USA). HT-29 cells with a density of 5 × 10^3^ and LS174T cells with a density of 7 × 10^3^ cells per well were seeded in 96-well plates. The absorbance optical (OD) density value was measured at 450 nm, using Multiskan FC (Thermo Scientific, Waltham, MA, USA), for 5 days after transfection.

### 2.14. Real-Time PCR

Total RNA was extracted by TRIzol (15596018, Invitrogen, Waltham, MA, USA), and 1 µg of RNA was reverse-transcribed into cDNA, using a ProtoScript First Strand cDNA Synthesis Kit (E6300L, NEW ENGLAND BioLabs, Ipswich, MA, USA). Quantitative expression analysis was performed with QuantiFast SYBR Green (204056, Qiagen, Hilden, Germany) with 20 ng of cDNA. *HPRT1* was used as a housekeeping gene. Primers of *MUC2* and *HPRT1* were from QuantiTect Primer Assay (QT00059066, QT01004675, Qiagen, Hilden, Germany). The detections were performed by Applied Biosystems 7300. The primer sequences for real-time PCR were *TRPM4* F 5′-TGGCTCTCACCTGCTTCCT-3′, R 5′-CCGCACCGTGAAAACCATG-3′; *CYBA* F 5′-ACCAGGAATTACTATGTTCGGGC-3′, R 5′-TAGGTAGATGCCGCTCGCAATG-3′; *MUC1* F 5′-CTGGTCTGTGTTCTGGTTGC-3′, R 5′-CCACTGCTGGGTTTGTGTAA-3′.

### 2.15. Western Blot

Protein lysates were prepared and quantified by using Pierce™ BCA Protein Assay Kit (Thermo Fisher Scientific, Waltham, MA, USA, #23225). NuPAGE™ 4–12% Bis-Tris Protein Gels and the respective running buffer (Thermo Fisher Scientific; Waltham, MA, USA, #NP0321PK2, #NP0001) were used for separation of 20 μg of each protein sample. Blotted membranes were blocked with 5% milk for 1 h, incubated overnight at 4 °C with the primary antibodies (HA tag monoclonal antibody (26183, Invitrogen, 1:5,000), anti-CYBA antibody (sc-271968, Santa Cruz, Heidelberg, Germany 1:250), anti-TRPM4 antibody (ab123936, Abcam, Cambridge, UK, 1:1000) and loading control alpha tubulin antibody (ab4074, Abcam, Cambridge, UK, 0.25 μ/mL) and subsequently for 1 h at room temperature with anti-rabbit IgG antibody (7074S, Cell Signaling Technology, Frankfurt, Germany, 1:10,000) and anti-mouse IgG antibody (7076S, Cell Signaling Technology, Frankfurt, Germany, 1:2500) diluted in 5% milk. The membranes were washed, and signals were detected by Immobilon Western Chemiluminescent HRP substrate (WBKLS0500, Millipore, Burlington, MA, USA) and scanned by INTAS Chemostar.

### 2.16. Immunofluorescence

Cells were seeded on Chamber slide (C6932, Sigma-Aldrich) with the seeding density of 30 × 10^3^ per well. The cells were allowed to attach to the wells overnight before transfecting with siRNA. After transfection with the siRNA for 72 h, slides were incubated with 4% paraformaldehyde (sc-281692, Santa Cruz, Heidelberg, Germany) 20 min at room temperature; PBS 5 min 3 times, 0.1% Triton X-100 (11332481001, Sigma-Aldrich, Saint Louis, MO, USA) 10 min at room temperature, PBS 5 min 3 times, 3% BSA block 1 h at 37 °C. After that, the slides were incubated with anti-MUC2 antibody (ab11197, Abcam, Cambridge, UK, 1:500) for 3 h at 37 °C, PBS 5 min 3 times, and with anti-mouse IgG (ab150113, Abcam, Cambridge, UK, 1:1000) for 1 h at 37 °C, PBS 5 min 3 times. The nucleus was dyed with 1 ug/mL DAPI (62248, Thermo Scientific) for 2 min at room temperature, PBS 2 min 3 times. The slides were mounted with Limonene Mounting Medium (ab104141, Abcam, Cambridge, UK,) and covered by coverslips (7695031, Th. Geyer, Renningen, Germany). Confocal microscopy was performed with Leica SP8, and images were saved as TIFF file formats. For each group in every experiment, we randomly selected three images with similar numbers of cells and uniform background.

### 2.17. Reactive Oxygen Species (ROS) Detection

Cellular ROS detection was performed with DCFDA Cellular ROS Detection Assay Kit (ab113851, Abcam, Cambridge, UK) in both HT-29 and LS174T cell lines, according to the manufacturer′s instructions.

### 2.18. Statistical Analysis

The comparison of proliferation, real-time PCR and ROS between the knockdown group and the control group were analyzed by non-paired Student’s *t*-test. Image J was used to calculate the fluorescence intensity and area in every group, and quantitative statistical comparison of fluorescence intensity of MUC2 was performed with non-paired Student’s *t*-test [32,33]. Statistical analysis was performed in GraphPad Prism 5.0. Significant differences were considered when *p* < 0.05. Means and standard errors of the means of each assay were presented on the graphs. For real-time PCR assays and ROS detection, we performed three independent experiments in triplicate for each analysis, and two for Western blot and immunofluorescence.

## 3. Results

### 3.1. Whole-Genome Sequencing Revealed No Variants in Known Colorectal Cancer Predisposing Genes

None of the 15 families had copy number variants or pathogenic or likely pathogenic variants or variants of unknown significance in any of the genes attributed to the hereditary CRC syndromes including *MLH1*, *MSH2*, *MSH6*, *PMS2*, *EPCAM*, *APC*, *MUTYH*, *STK11*, *PTEN*, *BMPR1A*, *SMAD4* and *TP53* nor in the genes suggested to be related with familial CRC including *POLE*, *POLD1*, *NTHL1*, *GERM1*, *GALNT12*, *CHEK2*, *BLM*, *AXIN2*, *ATM*, *BMPR1A*, *RECQL*, *RPS20, MLH3* and *MSH3*.

### 3.2. Identification of Loss-of-Function Variants by Whole-Genome Sequencing

**Figure 1:**
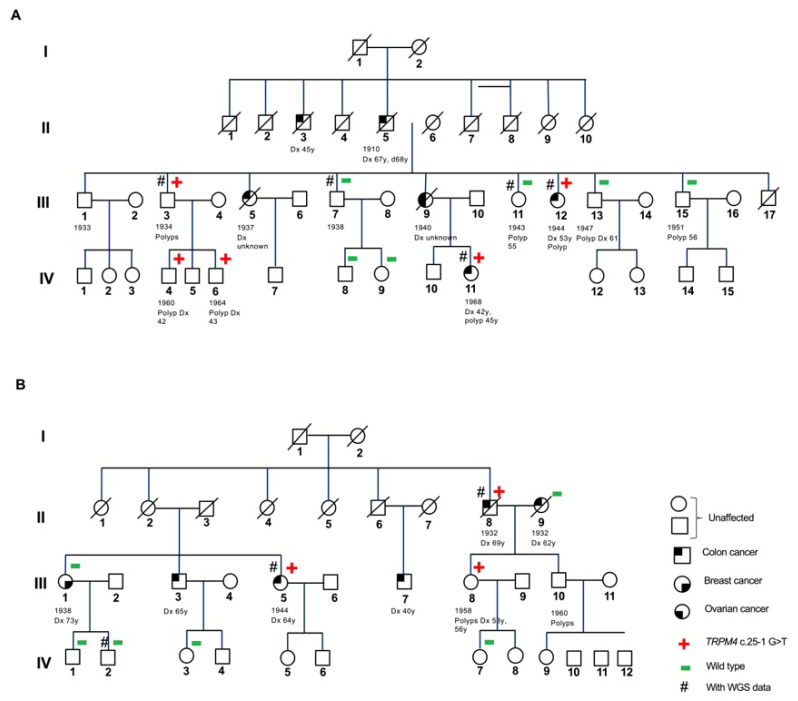
Pedigrees of colon cancer families. (**A**) Family 8 with CYBA c.246delC variant. (**B**) Family 11 with TRPM4 c.25-1 G > T variant.

We identified, altogether, 14 rare loss-of-function variants (nonsense variants, indels leading to a frameshift and variants in canonical splice sites) that segregated with the disease in 9 of the 15 whole-genome sequenced families (Supplementary Table S2). CADD scores of the variants were between 24.7 and 44, supporting their deleterious nature. The literature search indicated a role in inflammation and mucin homeostasis of the intestine for two genes, *CYBA* and *TRPM4,* that were mutated in two unrelated families (Figure 1); these were investigated further.

### 3.3. CYBA c.246delC and TRPM4 c.25-1 G > T Variant Segregation in CRC Families

In Family 8, we found a heterozygous germline *CYBA* c.246delC (ENST00000261623) variant, which segregated with the disease in the pedigree (Figure 1A and Figure S1, upper panel). In this family, four family members were diagnosed with CRC at ages from 42 to 67 years (II-5, III-9, III-12 and IV-11) and two with cancer in the abdominal cavity (II-3 and III-5). Several family members had also polyps in the colon. The two sequenced family members diagnosed with CRC (III-12 and IV-11) carried the variant, as did III-3, who was diagnosed with polyps in the colon and his two sons (IV-4 and IV-6) who were also diagnosed with polyps in their 40s. Of note, the mother of IV-11 (III-9) was diagnosed with both colon and ovarian cancer. In contrast, III-7 and both of his sons (IV-8 and IV-9) who did not carry the variant had no cancer, nor did III-13 or III-15, although they had polyps diagnosed at the age of 61 and 56 years.

In Family 11, a heterozygous germline *TRPM4* c.25-1 G > T (ENST00000252826) variant segregated with the disease in the pedigree (Figure 1B and Figure S1, lower panel). In this family, two siblings (III-3 and III-5), their cousin (III-7) and their uncle (II-8) were diagnosed with CRC at the age ranging from 40 to 69 years. Several family members had also polyps. The two sequenced family members (II-8 and III-5) diagnosed with CRC carried the variant, as did the daughter of II-8 (III-8), who had multiple polyps. Of note, the wife of II-8 (II-9), who was also diagnosed with CRC, did not carry the variant. No *TRPM4* c.25-1 G > T variant was found in a female member (III-1) diagnosed with breast cancer at the age of 73 years, whose colonoscopy was negative until the age of 74 years, as with her two sons (IV-1 and IV-2).

### 3.4. CYBA c.246delC and TRPM4 c.25-1 G > T Are Rare Variants

*CYBA* c.246delC is absent from the population-based variant databases gnomAD, including data from 76156 individuals from the main world populations (Available online: https://gnomad.broadinstitute.org/ (accessed on 17 January 2022)) in the Exome Variant Server, including data from 6503 individuals from European and African American populations (Available online: http://evs.gs.washington.edu/EVS (accessed on 17 January 2022)) and Leiden Open Variation Database (LOVD; Available online: https://www.lovd.nl/ (accessed on 17 January 2022)), but it was reported in dbSNP (hg38) (Available online: http://www.ncbi.nlm.nih.gov/snp (accessed on 17 January 2022)), showing an allele frequency of 0.00004 (5/125,568, Available online: https://www.ncbi.nlm.nih.gov/snp/rs1439134665 (accessed on 17 January 2022)) and Clinvar (Available online: https://www.ncbi.nlm.nih.gov/clinvar/ (accessed on 17 January 2022)), in which it was suggested by a single submitter to be an apathogenic variant of granulomatous disease (Available online: https://www.ncbi.nlm.nih.gov/clinvar/variation/619030/ (accessed on 17 January 2022)). *TRPM4* c.25-1 G > T is absent in all of the above population databases.

### 3.5. Screening of a Large Cohort of Familial CRC Patients

Screening of the *CYBA* c.246delC and *TRPM4* c.25-1 G > T variants in 1705 familial CRC cases and 1674 healthy elderly individuals, both from Poland, using custom-made Taqman assays, confirmed the presence of the CYBA variant in Family 8 and identified the *CYBA* c.246delC variant in four additional familial CRC cases and two healthy individuals (odds ratio 2.46, 95% confidence interval 0.48–12.69). Two of the CRC patients, diagnosed at ages of 59 years and 64 years, had a family history of CRC. One CRC patient, diagnosed at the age of 44 years, had no contact to family members, and another one, diagnosed at the age of 63 years, had a family history of female genital tract and larynx cancers. The sampling ages of the two healthy individuals were 94 years and 69 years, and they had no family history of any cancer. The existence of the heterozygous *CYBA* c.246delC variant was confirmed by Sanger sequencing in all positive samples. In addition to the *TRPM4* variant in Family 11, no other families were found to carry the variant.

### 3.6. CYBA c.246delC Led to Loss of CYBA Protein

**Figure 2:**
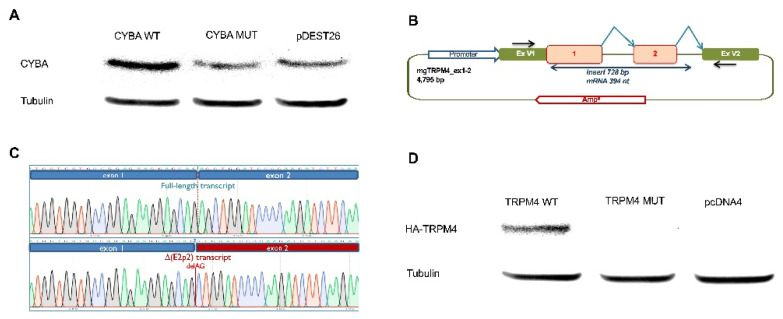
*CYBA* c.246delC and *TRPM4* c.25-1G > T variants led to loss of the corresponding proteins. (**A**) Western blot detection of the CYBA protein in HEK293T transfected with wild-type (CYBA WT), mutated (CYBA MUT) or control plasmids (pDEST26). HEK293T cells transfected with the mutant CYBA plasmid expressed less protein compared to cells transfected with the wild-type CYBA plasmid. CYBA c.246delC-transfected cells expressed a similar amount of protein to those transfected with pDEST26 only. (**B**) Outline of the *TRPM4* minigene construct. The black arrows in vector exons V1 and V2 indicate specific RT-PCR minigene primers; broken arrows represent the expected splicing reactions. (**C**) Sequencing traces of the transcripts generated by the wild-type (above) and mutant (c.25-1G > T) minigenes, suggesting that TRPM4 c.25-1G > T led to a frameshift transcript r.25_26del, that would be equivalent to c.25delAG. (**D**) *TRPM4* c.25delAG led to the loss of TRPM4 protein. Western blot of TRPM3-HA Tag antibody in HEK293T cells transfected with wild-type, mutated or control plasmids. HEK293T cells transfected with pcDNA4TO-HA-TRPM4 c.25delAG (TRPM4 MUT) and pcDNA4 did not express any HA-TRPM4; only HEK293T cells transfected with wild-type pcDNA4TO-HA-TRPM4 (TRPM4 WT)-expressed HA-TRPM4.

As shown in Figure 2A, HEK293T cells transfected with pDEST26-*CYBA* c.246delC expressed less CYBA protein than those transfected with wild-type pDEST26-*CYBA*. Moreover, pDEST26 only expressed a similar amount of CYBA to that of the mutant, suggesting that *CYBA* c.246delC led to a loss of the CYBA protein.

### 3.7. TRPM4 c.25-1G > T Led to a Frameshift Transcript r.25_26del and Loss of TRPM4 Protein

The in silico analysis of the *TRPM4* wild type and c.25-1G > T sequences with MaxEnt Scan (MES) showed that this variant disrupted the canonical acceptor site of TRPM4 exon 2 (MES = 11.38 → 2.78) but simultaneously created a strong de novo acceptor site two nucleotides downstream (MES = 9.9).

The minigene mgTRPM4_ex1-2 was constructed by using DNA from a patient (III-5). The wild-type and mutant minigenes were introduced into MCF-7 cells, and RNA was isolated and reverse transcribed (Figure 2B). The wild-type minigene produced a single transcript of the expected size (394 nt) and structure (V1- TRPM4 ex1-2 –V2). The c.25-1G>T-minigene also generated a single transcript whose sequence revealed the loss of the first two nucleotides of exon 2 (r.25_26del), using the new alternative acceptor site predicted by MES. This transcript was called ∆(E2p2) by following previously suggested rules [34]. Moreover, ∆(E2p2) would introduce a frameshift and a premature protein truncation 17 codons downstream (p. Ser9Leufs*17) that would inactivate the TRPM4 protein. High-resolution fragment analysis of fluorescent RT-PCR products confirmed the presence of this aberrant transcript (Figure 2C and Figure S2).

As *TRPM4* c.25-1G > T was not in the cDNA, and the minigene assay showed that *TRPM4* c.25-1G>T led to r.25_26del., i.e., equivalent of c.25delAG, we created pcDNATO-HA-*TRPM4* c.25delAG. We transfected the pcDNATO-HA-*TRPM4* c.25delAG into HEK293T cells to see if the variant affects the expression of TRPM4. As shown in Figure 2D, only HEK293T cells transfected with wild-type pcDNA4TO-HA-*TRPM4*-expressed HA-TRPM4, while HEK293T cells transfected with pcDNA4TO-HA-*TRPM4* c.25delAG or pcDNA4 did not express HA-TRPM4, confirming that *TRPM4* c.25delAG resulted from *TRPM4* c.25-1G > T led to a loss of TRPM4 protein.

### 3.8. Knockdown of CYBA or TRPM4 Promoted Proliferation in HT-29 Cells

**Figure 3:**
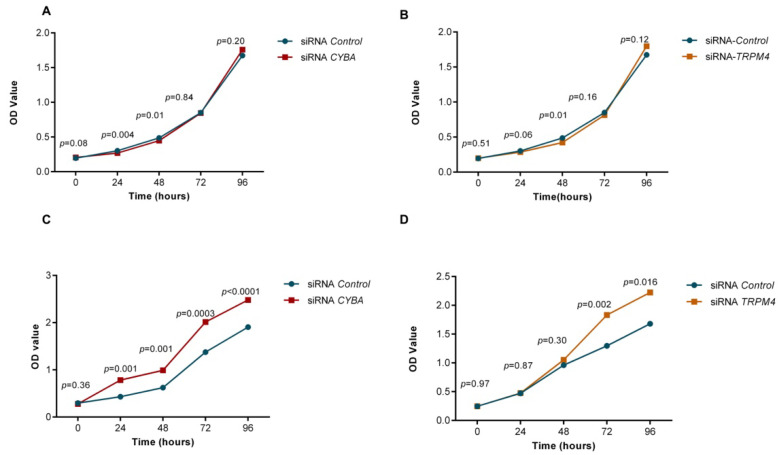
Effect of CYBA or TRPM4 knockdown on cell proliferation of LS174T and HT29 cells measured by CCK-8 cell assay. (A) LS174T cells transfected with siRNA-CYBA (**A**) or siRNA-TRPM4 (**B**) showed similar proliferation compared to siRNA-control transfected cells. HT29 cells transfected with siRNA-CYBA (**C**) or siRNA-TRPM4 (**D**) showed a significant increase in proliferation compared to siRNA-control transfected cells.

To study the effect of *CYBA* and *TRPM4* on cell proliferation, we knocked down *CYBA* and *TRPM4* with respective siRNAs, separately, in two colorectal adenocarcinoma cell lines, LS174T and HT-29; LS174T is a mucin secreting cell line. Moreover, qPCR and Western blot analysis confirmed the absence of CYBA or TRPM4 mRNA and protein expression in both LS174T and HT-29 cells (Supplementary Figure S3A–D). While knockdown of *CYBA* or *TRPM4* did not seem to affect cell proliferation of LS174T cells in in vitro cell proliferation assay (Figure 3A,B), knockdown of *CYBA* or *TRPM4* in the HT-29 cells led to an increase in cell proliferation throughout the time up to 96 h (Figure 3C,D; *p* < 0.05).

### 3.9. Knockdown of TRPM4 Decreased MUC1 and MUC2 in Both LS174T and HT-29 Cells

**Figure 4:**
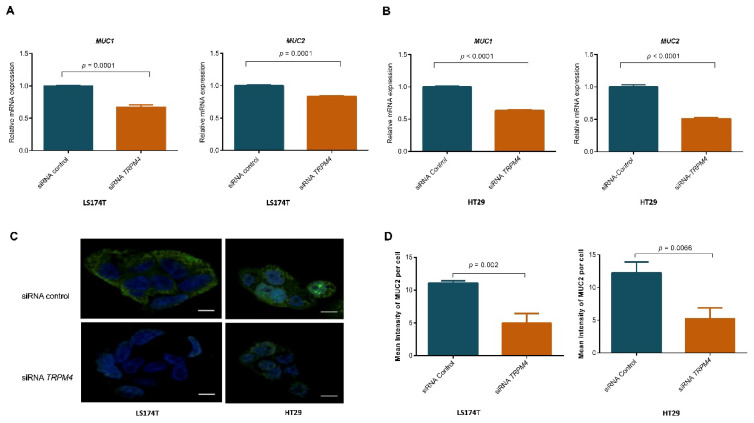
RT-PCR results showed that mRNA levels of *MUC1* and *MUC2* decreased after siRNA knockdown of *TRPM4* in LS174T (**A**) and HT-29 (**B**) cells. Three independent experiments in triplicate were performed, and means and standard errors of the means are presented on the graphs. A clear decrease of MUC2 expression is seen in immunofluorescence after knockdown of *TRPM4*, especially in mucin-secreting cell line LS174T (**C**), but also in HT-29 (**D**) cells. Two independent experiments were performed, and means and standard errors of the means are presented on the graphs.

In order to test the hypothesis that loss of *TRPM4* disrupts the mucus barrier by inhibiting mucin secretion, we measured the expression of *MUC1* and *MUC2* by qPCR after downregulating *TRPM4* in LS174T and HT-29 cell lines. The mRNA levels of *MUC1* and *MUC2* were decreased after knockdown *TRPM4* in both LS174T and HT-29 cells (*p* ≤ 0.0001; Figure 4A,B). Moreover, the protein expression of MUC2, measured by immunofluorescence was decreased in both LS174T and HT-29 cells after the knockdown of *TRPM4* compared to those treated with control siRNA (LS174T: *p* = 0.002; HT-29: *p* = 0.006; Figure 4C,D).

### 3.10. Knockdown of CYBA or TRPM4 Led to ROS Deficiency

**Figure 5:**
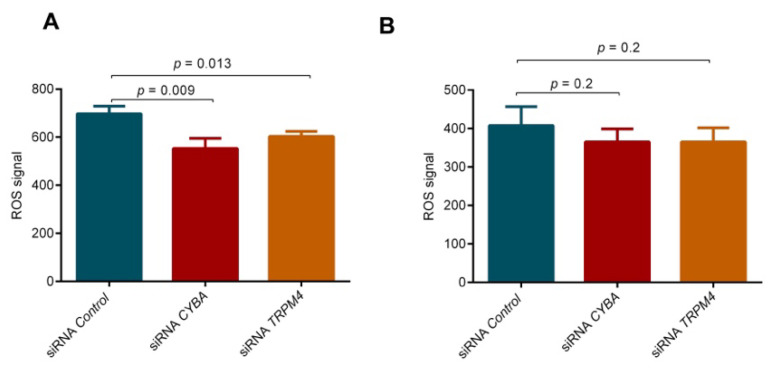
ROS activity upon *CYBA* or *TRPM4* knockdown. *CYBA* or *TRPM4* depletion led to significant reduction of ROS in LS174T cells (**A**), but no decrease in HT-29 cells (**B**). Three independent experiments in triplicate were performed, and means and standard errors of the means are presented on the graphs.

As *CYBA* is involved in ROS modulation, we measured the ROS activity in both LS174T and HT-29 cells after siRNA-mediated knockdown of *CYBA* or *TRPM4*. There was a small, but significant decrease in ROS activity in LS174T cells after the knockdown of *CYBA* or *TRPM4* (*p* = 0.009 and 0.014; Figure 5A), but no decrease in HT-29 cells (Figure 5B).

## 4. Discussion

We carried out germline WGS analyses of 15 Polish CRC families, each of which presented with several CRCs. Loss-of-function variant analysis in two separate families pointed out two rare variants in the genes *CYBA* and *TRPM4* that segregated with the disease in these families. The variant in *CYBA* (*CYBA* c.246delC) leads to a frameshift and a premature protein truncation 41 codons downstream (p.Phe83Asnfs*41), while the splice site variant in *TRPM4* (*TRPM4* c.25-1 G > T) leads to a frameshift and a premature protein truncation 17 codons downstream (p. Ser9Leufs*17). We showed that both variants lead to a loss of protein expression. *CYBA* encodes the alpha chain of cytochrome B-245, which is part of the NADPH oxidase (NOX) system, to generate superoxide [35]. It is a component in NOX1–4, of which NOX1 is expressed in the gastrointestinal tract and NOX2 in phagocytes [36]. *TRPM4* encodes a nonselective monovalent cation channel, the upregulation of which enhances sodium entry, which, in turn, leads to depolarization of the membrane potential [37]. Both genes are highly expressed in the colon and are active in mucin-secreting goblet cells [38,39]. Although the functions of these two genes appeared initially to be quite diverse, our literature search resulted in a challenging combination of pathways targeting inflammatory bowel disease, a risk factor of CRC [14]. The present study gives further insight into the complementary function of the *CYBA* and *TRPM4* pathways, which converge in the protection of the colonic epithelium. The colon is covered by an inner colon mucus layer that is proteolytically converted into an outer mucus layer, both consisting of the MUC2 mucin as the main protein component [40]. The inner mucus layer acts as a barrier against bacteria; only when it is damaged are bacteria able to reach the epithelial surface, which may lead to severe inflammation [40].

Superoxide, generated by NOX analogues, is a member of ROS for which the optimal concentration is critical; overproduction will lead to oxidative stress and development of disease, and, likewise, insufficient ROS production may be detrimental to health [38]. Germline variants in *CYBA* are associated with autosomal recessive chronic granulomatous disease, characterized by the failure of activated phagocytes (neutrophils and macrophages), to generate enough superoxide to accomplice intracellular killing of pathogens. Patients suffer from life-threatening infections and from excessive inflammatory reactions [41]. About half of chronic granulomatous disease colon patients present with granuloma formation and acute or chronic inflammation mimicking inflammatory bowel disease [42]. Germline *CYBA* variants have been associated with early onset inflammatory bowel disease [43,44]. Bacterial penetration of colonic epithelium, the normally restricted zone, is observed in many colitis models and also in patients with inflammatory bowel disease, such as ulcerative colitis [45]. Mucus defects that allow bacteria to reach the epithelium and to stimulate an immune response can lead to the development of intestinal inflammation [46]. The comparison of inflamed ulcerative colitis patients and *MUC2*^−/−^ mice revealed that bacteria in both models had been able to penetrate the colon epithelium, thus causing inflammation [46]. *MUC2*^−/−^ mice have also been shown to develop adenomas in the intestine, the majority of which even progressed into adenocarcinomas [47].

**Figure 6:**
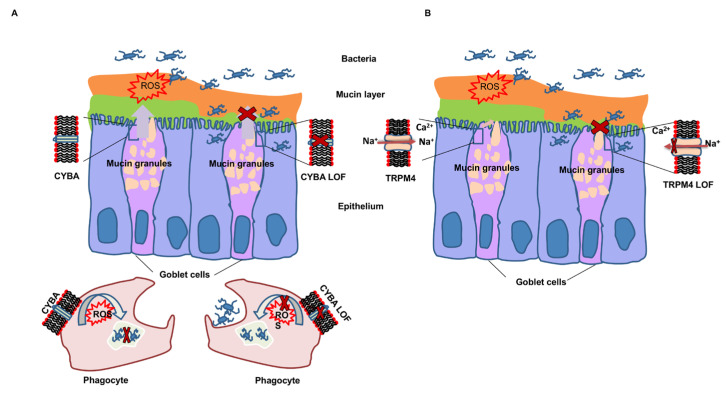
Schematic presentation of the suggested consequences of *CYBA* and *TRPM4* variants on colonic mucin layer integrity. (**A**) The loss-of-function variant in *CYBA* leads to decreased ROS production and promotes CRC by faltering defense against intestinal bacteria at colonic epithelium and by suppressing bacterial killing by intestinal phagocytes. (**B**) The loss-of-function variant in *TRPM4* leads to decreased mucus secretion due to inactivation of the TRPM4 channel, potentially leading to mucus-layer disruption with bacterial penetration into the mucin-protected epithelium, inflammation and colorectal cancer. The function of the wild-type CYBA and TRPM4 is shown in the left goblet cell; the function of CYBA and TRPM4 after the loss-of-function (LOF) variant is shown in the right goblet cell.

An important clue about the mechanism of action of *CYBA* in inflammatory bowel disease was recently shown in *CYBA* mutant mice which generated low intestinal ROS [48]. The mice suffered from profound mucus layer disruption, with bacterial penetration into crypts, and from a compromised innate immune response to invading microbes, leading to mortality. The results implicated the loss of the mucus barrier and innate immune defense in the pathogenesis of intestinal inflammation, due to ROS deficiency [48]. In another study, using a dextran sodium sulfate–induced colitis mouse model, ROS deficient Ncf1-mutant mice developed well-differentiated adenocarcinomas, while only high-grade dysplasia without malignant invasion was observed in ROS-proficient mice with Ncf1 wild type, suggesting that ROS deficiency may cause CRC in response to environmental risk factors [49]. Our experimental results and data from the literature suggest that the loss-of function variant in *CYBA* promotes CRC by at least two mechanisms because of depressed ROS production, first by faltering defense against intestinal bacteria at colonic epithelium and second by suppressing bacterial killing by intestinal phagocytes, such as neutrophils and macrophages (Figure 6A) [38].

*TRPM4* encodes a calcium-activated nonselective ion channel, the activity of which increases with increasing intracellular calcium concentration; however, this channel does not transport calcium. Disturbance of the membrane potential is deleterious to calcium homeostasis, and this is suggested to contribute to carcinogenesis through uncontrolled cell migration and invasion [37]. The *TRPM4* gene is highly expressed in CRC tumor buds, contributing to the proliferation and invasion of tumor cells [50]. TRPM4 cooperates in the control of mucin secretion from goblet cells in response to extracellular stimuli [39]. The results in the human colonic cancer goblet cell line (HT-29-18N2) showed that TRPM4 protein controls calcium-mediated secretion of MUC2 and MUC5AC in conjunction with a Na^+^/Ca^2+^ exchanger NCX [39]. Knockdown of TRPM4 in the HT29-18N2 cells blocked MUC2 secretion [39]. In support of this finding, we showed for the first time that *TRPM4* knockdown also affects *MUC2* mRNA and protein expression. These findings suggest that the loss-of-function variant in *TRPM4* leads to disruption of the mucus layer, allowing bacterial penetration into the mucin-protected epithelium, and thus resulting in inflammation and risk of CRC (Figure 6B). In their publication, Cantero-Recasens et. al. speculated about two distinct modes of mucin secretion: In baseline mucin secretion, TRPM4 is inactive and mucin secretion is regulated by KChIP3 and intracellular Ca^2+^ oscillation [39]. Stimulated mucin secretion is caused by exogenous stimulation of the cells by, for example, ATP or IL-13, which leads to a rapid Ca^2+^ release from the endoplasmic reticulum, activation of TRPM4 and increase of intracellular Na^+^. This, in turn, triggers NCX to export Na^+^ and import Ca^2+^, leading to a rapid burst in mucin secretion. The above authors showed similar results in goblet cells earlier with TRPM5, a homologue of TRPM4 [51]. *TRPM4* and *TRPM5* share high sequence homology and similar biophysical properties, yet they are not able to fully compensate each other [51]. In addition, by affecting mucin expression and secretion, TRPM4 may influence inflammation through the modulation of immune responses [31,37]. As an alternative mechanism, TRPM4 has been implicated in the regulation of the Wnt signaling pathway, which is often dysregulated in CRC [37].

The most commonly mutated gene in CRC is *KRAS*. When mutated, oncogenic RAS-mediated signaling cascades not only drive tumor initiation, maintenance and progression, but also affect cellular metabolism, including ROS signaling [52]. While mutated *KRAS* reportedly is leading to increased ROS generation and tumor promotion, elevated ROS levels may also trigger tumor cell death. In our study, we observed a cell-line-dependent decrease in ROS generation and cell proliferation after the knockdown of *CYBA* or *TRPM4*. Thus, the effect on ROS generation and downstream signaling may be context dependent and requires further research [37]. On the other hand, oncogenic RAS signaling may promote tumorigenesis, both when the MUC2 production is suppressed or enhanced and depending on the specific subtype of CRC [53]. Unfortunately, no tumor sample or tumor pathology data were available from the CRC families to analyze the type of tumors of the patients.

The present variants were not present in the gnomAD database, which listed eight other loss-of-function variants for *CYBA* and 54 for *TRPM4*; the database covers 125,748 exome sequences. Of note, exactly the same *CYBA* c.246delC variant was submitted to the Clinvar database as a pathogenic variant of granulomatous disease, which is a recessive condition (Available online: https://www.ncbi.nlm.nih.gov/clinvar/variation/619030/ (accessed on 17 January 2022)). In the CRC family, the variant was present in the heterozygous form, and the phenotype would be expected to be milder with a later onset than in the case of granulomatous disease. Unfortunately, tumor samples from the patients were not available for testing this. Although the *CYBA* variant was absent from 125,748 exomes of the gnomAD database, it was found in four additional familial CRC patients among 1705 tested Polish patients and in 2/1674 controls, of whom only one was past the common diagnostic age of CRC. As a DNA sample was only available for the index case of the four families, we could not evaluate the segregation of the variant with the disease in these families, and this was a limitation of our study.

The Polish National Colorectal Cancer Screening program offers colonoscopy to all inhabitants between 50 and 66 years; individuals with family history of CRC are eligible starting at the age of 40 years. Among 236,089 individuals screened from 2000 to 2011, 17.7% were diagnosed with adenomas; among individuals with a family history, 18.9% [54]. As individuals with adenomas are at an increased risk of CRC, we considered family members diagnosed with polyps as potential carriers of the predisposing variant. Since we did not have access to any clinical or pathological features of the patients, their tumors and polyps, we were not able to evaluate the risk category of these individuals. Thus, we can only speculate about the role of inflammation in the patients carrying the variant.

In the present study, we focused on the loss-of-function variants, as these are considered to be the most deleterious types of sequence variants. We plan to assess the less deleterious variants, such as missense variants in the future, but they require an increased focus on functional characterization. Many biological pathways are cell and tissue specific, thus calling for the application of different cell lines. In the present study, MUC1 and MUC2 secretion was affected in both LS174T and HT-29 cell lines, while proliferation was affected only in the HT-29 cells, and the effect on ROS activity was more prominent in the LS174T cells.

## 5. Conclusions

Our germline sequencing efforts of familial CRC led to the identification of two possible pathogenic variants of two likely CRC-predisposing genes *CYBA* and *TRPM4*. The function of these two genes appeared to provide complementary pathways of protecting the colonic epithelium, as shown in Figure 6 [40]. Although our data did not allow us to demonstrate a direct mechanism to cancer formation, we showed associations of the *CYBA* and *TRPM4* functions with ROS and mucin production with a likely link to tumorigenesis of CRC.

## Data Availability

The WGS data generated in this study are available in EGA under accession number EGAS00001005118. Other data that support the findings of this study are available from the corresponding author upon request.

## Supplementary Materials

The following supporting information can be downloaded at: https://www.mdpi.com/article/10.3390/cancers14030670/s1, Figure S1. Sanger sequencing results of the CYBA (upper panel) and TRPM4 (lower panel) wild type and mutant sequences; Figure S2. Splicing functional assays of TRPM4 variant c.25-1G>T. (A) Fluorescent fragment analysis by capillary electrophoresis of RT-PCR products generated by the wild type and mutant minigenes. The canonical and the aberrant ∆(E2p2) transcripts are shown as blue and red peaks, respectively, while the LIZ500 size standard is displayed as orange peaks. (B) Schematic representation of the canonical (above) and anomalous (below) transcripts. Variant c.25-1G>T is shown in red; acceptor sites are underlined; splicing events are shown as broken arrows; Figure S3. SiRNA knockdown of CYBA or TRPM4 lead to reduced mRNA and protein of the respective genes: mRNA (A) and protein (B) levels of CYBA and TRPM4 decreased after siRNA knockdown of CYBA or TRPM4 in LS174T cells; mRNA (C) and protein (D) levels of CYBA and TRPM4, decreased after siRNA knockdown of CYBA or TRPM4 in HT-29 cells. For real-time PCR assays, three independent experiments in triplicate were performed and means and standard errors of the means are presented on the graphs. For western blot two independent experiments were performed;Table S1. Summary of the sequencing coverage and quality statistics for each sample; Table S2. Loss-of-function variants segregated with the disease in 15 colorectal cancer families.
