# Supplementary material for: Germline Variants of CYBA and TRPM4 Predispose to Familial Colorectal Cancer
Source: Cancers (Basel). 2022 Jan 28;14(3):670. doi: 10.3390/cancers14030670 (PMC8833488; doi:10.3390/cancers14030670)
Supplement: Supplementary file 1 [file cancers-14-00670-s001.zip › Supplementary Material.pdf]

## Supplementary material

Table S1. Summary of the sequencing coverage and quality statistics for each sample.

Table S2. Loss-of-function variants segregated with the disease in 15 colorectal cancer families.

Figure S1. Sanger sequencing results of the *CYBA* (upper panel) and *TRPM4* (lower panel) wild type and mutant sequences.

Figure S2. Splicing functional assays of *TRPM4* variant c.25-1G>T. (A) Fluorescent fragment analysis by capillary electrophoresis of RT-PCR products generated by the wild type and mutant minigenes. The canonical and the aberrant Δ(E2p2) transcripts are shown as blue and red peaks, respectively, while the LIZ500 size standard is displayed as orange peaks. (B) Schematic representation of the canonical (above) and anomalous (below) transcripts. Variant c.25-1G>T is shown in red; acceptor sites are underlined; splicing events are shown as broken arrows.

Figure S3. SiRNA knockdown of CYBA or TRPM4 lead to reduced mRNA and protein of the respective genes: mRNA (A) and protein (B) levels of CYBA and TRPM4 decreased after siRNA knockdown of CYBA or TRPM4 in LS174T cells; mRNA (C) and protein (D) levels of CYBA and TRPM4, decreased after siRNA knockdown of CYBA or TRPM4 in HT-29 cells. For real-time PCR assays, three independent experiments in triplicate were performed and means and standard errors of the means are presented on the graphs. For western blot two independent experiments were performed.
