# Supplementary material for: Germline Variants of CYBA and TRPM4 Predispose to Familial Colorectal Cancer
Source: Cancers (Basel). 2022 Jan 28;14(3):670. doi: 10.3390/cancers14030670 (PMC8833488; doi:10.3390/cancers14030670)
Supplement: Supplementary file 1 [file cancers-14-00670-s001.zip › Supplementary Table 2.pdf]

Supplementary Table S2. Loss-of-function variants segregated with the disease in 15 colorectal cancer families.

Family_ID	Gene	Gene name	Chrom_Pos_Ref_Alt	Loss-of-function variation	Effect*	CADD_PHRED_score	Function
**Family_4**	ARRDC4	arrestin domain containing 4	15_98512490_C_T	stopgain SNV	p.Arg255Ter	44	protein ubiquitination, glucose metabolism, innate immunity
**Family_7**	ZNF471	zinc finger protein 471	19_57036072_C_CAA	frameshift insertion	p.Thr214LysfsTer176	35	transcription regulation
**Family_7**	HEXB	hexosaminidase B (beta polypeptide)	5_73989526_C_T	stopgain SNV	p.Arg170Ter	37	degradation of GM2 gangliosides
**Family_8**	**CYBA**	**cytochrome b-245, alpha polypeptide**	**16_88713203_AG_A**	**frameshift deletion**	**p.Phe83AsnfsTer41**	**25.3**	**superoxide generation**
**Family_9**	ZNF408	zinc finger protein 408	11_46726628_C_T	stopgain SNV	p.Gln460Ter	36	transcription regulation in retina
**Family_11**	**TRPM4**	**transient receptor potential cation channel subfamily M member 4**	**19_49661448_G_T**	**splicing**	**c.25-1 G>T p.Ser9LeufsTer17**	**26.1**	**calcium-activated nonselective ion channel, regulation of mucin secretion**
**Family_12**	LGALS3BP	lectin, galactoside-binding, soluble, 3 binding protein	17_76969123_C_T	stopgain SNV	p.Trp186Ter	35	integrin-mediated cell adhesion, immune response
**Family_12**	SEC23IP	SEC23 Interacting Protein	10_121685740_G_T	splicing	c.2313+1G>T	24.7	endoplasmic reticulum to Golgi vesicle-mediated transport
**Family_13**	GALNT5	UDP-N-acetyl-alpha-D-galactosamine:polypeptide N-acetylgalactosaminyltransferase 5 (GalNAc-T5)	2_158157422_C_T	stopgain SNV	p.Arg784Ter	44	mucin-type O-glycosylation of Golgi proteins
**Family_13**	ABCA8	ATP-binding cassette, sub-family A (ABC1), member 8	17_66887690_C_T	stopgain SNV	p.Trp988Ter	36	ATP-dependent lipophilic drug transporter
**Family_14**	SEPT12	septin 12	16_4833555_G_A	stopgain SNV	p.Gln212Ter	37	filament-forming cytoskeletal GTPase, involved in spermatogenesis
**Family_14**	POLN	polymerase (DNA directed) nu	4_2230895_G_A	stopgain SNV	p.Gln22Ter	36	DNA repair and homologous recombination
**Family_15**	C1orf177	LEXM, lymphocyte expansion molecule	1_55272689_AT_A	frameshift deletion	p.Asn44ThrfsTer24	24.8	
**Family_15**	ESD	esterase D	13_47365507_TC_T	frameshift deletion	p.Gly14AspfsTer13	35	serine hydrolase involved in the detoxification of formaldehyde

* nomenclature according to Human Genome Structural Variation Consortium in the Ensembl canonical transcript
