# Supplementary figures and images for: Germline Variants of CYBA and TRPM4 Predispose to Familial Colorectal Cancer
Source: Cancers (Basel). 2022 Jan 28;14(3):670. doi: 10.3390/cancers14030670 (PMC8833488; doi:10.3390/cancers14030670)

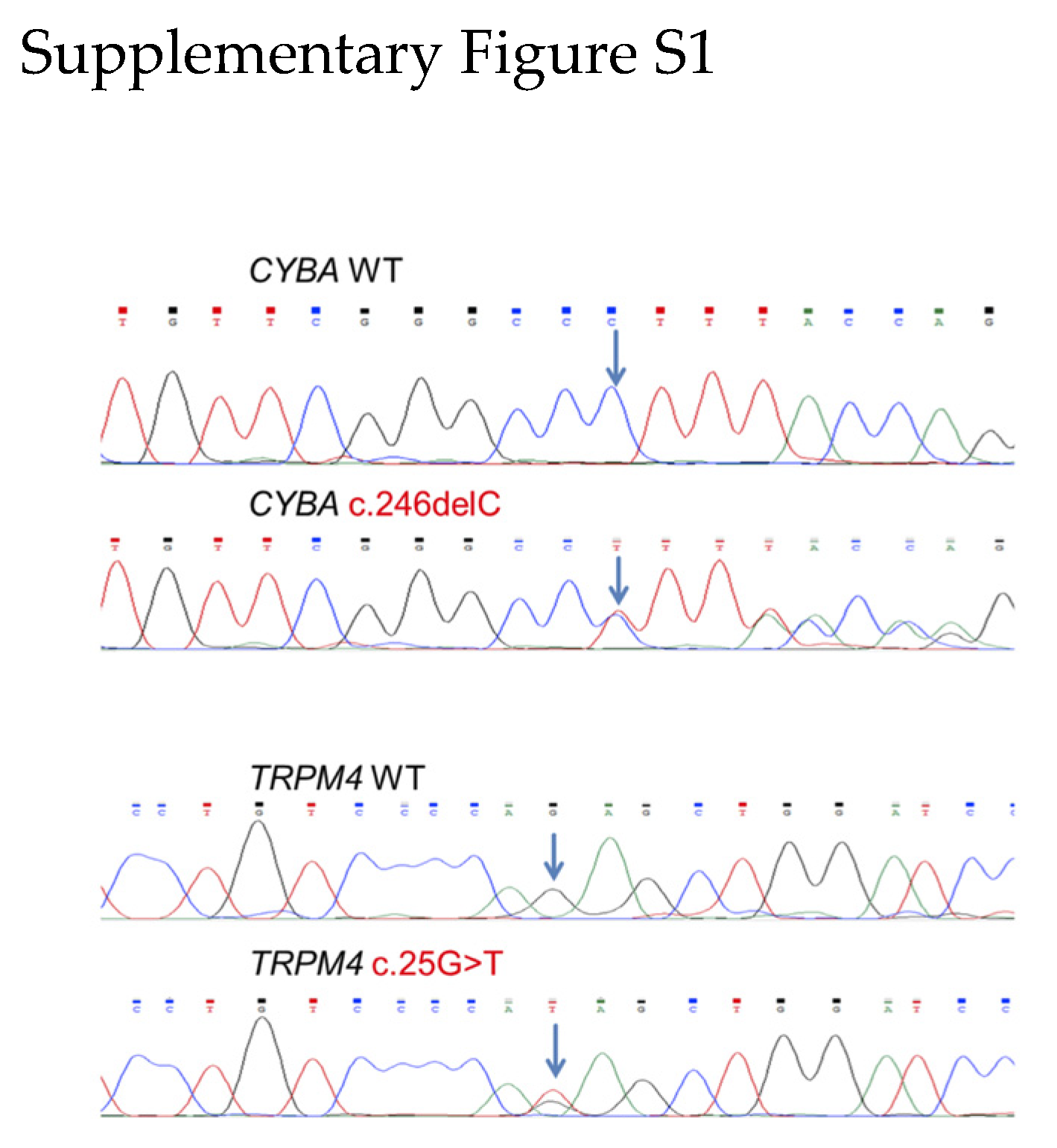

Supplement: Supplementary file 1 [file cancers-14-00670-s001.zip › Supplementary Figure S1.tif]

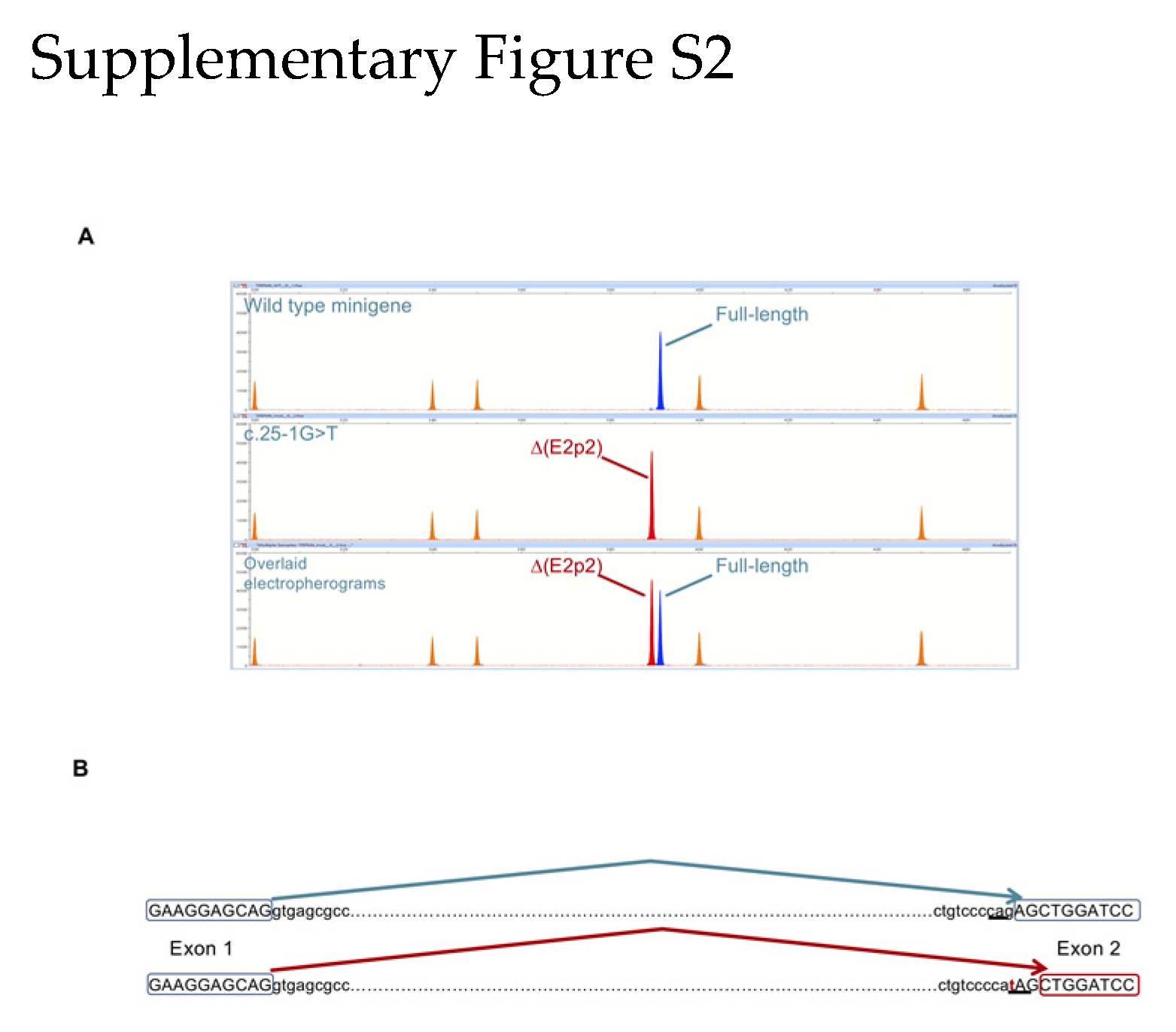

Supplement: Supplementary file 1 [file cancers-14-00670-s001.zip › Supplementary Figure S2.tif]

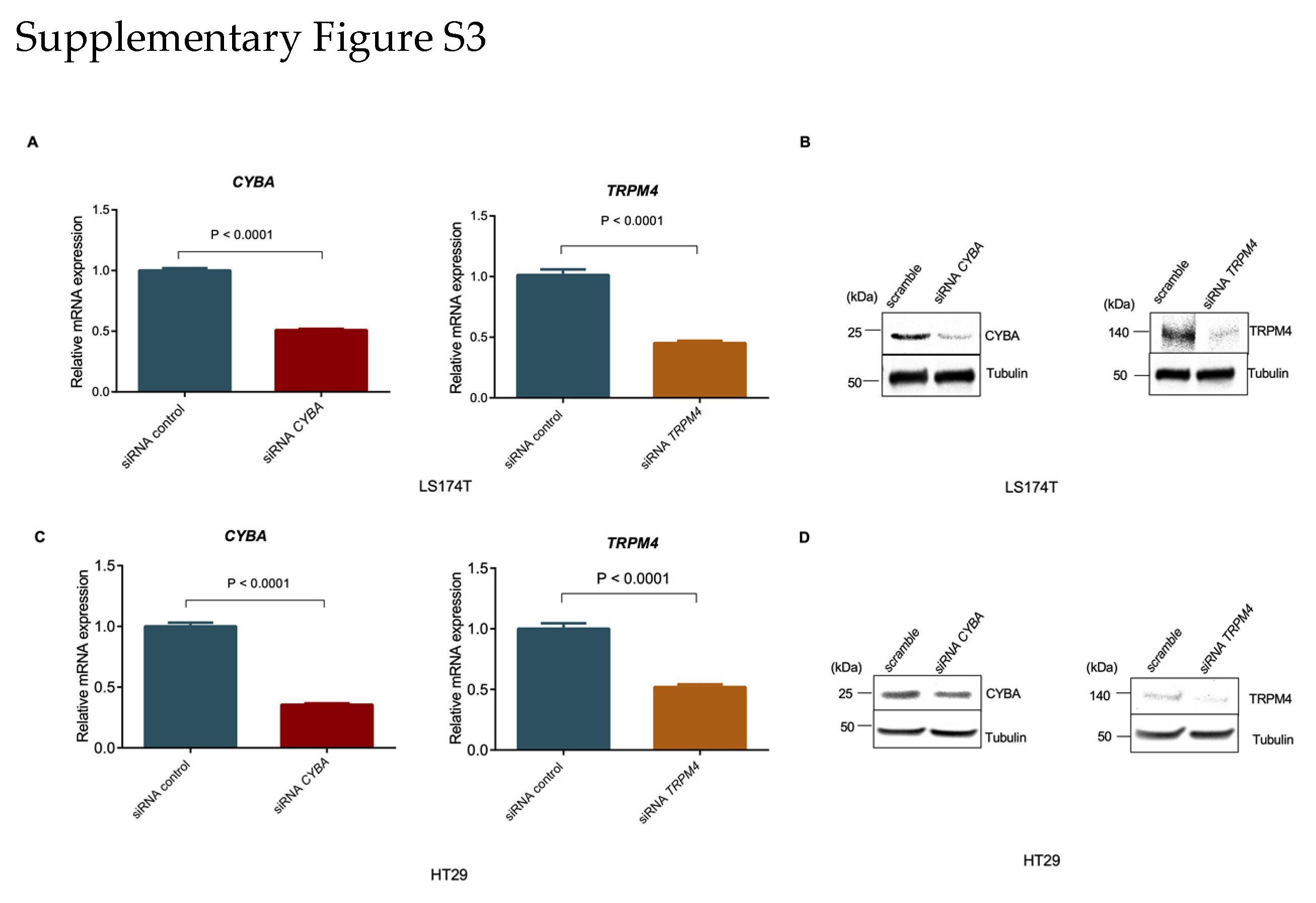

Supplement: Supplementary file 1 [file cancers-14-00670-s001.zip › Supplementary Figure S3.tif]
